# Behavioral choices and enabling conditions for implementing independence-supportive care among nursing assistants in residential aged care facilities in Hunan Province, China: a qualitative study

**DOI:** 10.3389/fpubh.2026.1972560

**Published:** 2026-10-06

**Authors:** Honghu Chen, Xuan Cheng, Jiayu Hou, Jiayu Liao, Weihong Wang

**Affiliations:** 1 School of Nursing, Hunan Normal University, Changsha, Hunan, China; 2 Kiang Wu Nursing College of Macau, Macao, China

**Keywords:** COM-B model, healthy aging, independence-supportive care, nursing assistants, older adults, qualitative study, residential aged care facilities

## Abstract

**Objective:**

To explore factors influencing nursing assistants’ implementation of independence-supportive care in nursing homes.

**Methods:**

A descriptive qualitative study was conducted using purposive sampling. From November 2025 to February 2026, 12 nursing assistants from five nursing homes in Hunan Province, China, participated in semi-structured interviews. The interview data were analyzed using a practical, codebook-oriented thematic analysis approach and subsequently interpreted using the Capability, Opportunity, Motivation–Behavior model.

**Results:**

Five themes were identified: (1) knowledge and skills related to independence-supportive care remained unstandardized; (2) residents’ functional status, willingness, and expectations of care affected their participation; (3) poor integration between assessment and care planning prevented retained abilities from becoming daily care goals; (4) limited staffing, time, and assistive devices led nursing assistants to take over residents’ tasks; and (5) safety risks and accountability pressures promoted risk-averse care. Although nursing assistants used some independence-supportive practices in daily work, these practices were largely based on personal experience and lacked standardized criteria for assessing task-specific abilities and determining appropriate levels of assistance.

**Conclusion:**

Nursing assistants’ implementation of independence-supportive care is shaped by the interaction of capability, opportunity, and motivation. Residential aged care facilities may consider providing activity-specific training, incorporating retained abilities and graded assistance into care plans, strengthening interdisciplinary collaboration, improving staffing and access to assistive devices, and establishing clear risk-management and shared-accountability mechanisms. Residents and families should also be informed that independence-supportive care is intended to maintain functional abilities rather than reduce the amount or quality of care. The effectiveness of these proposed strategies requires evaluation in future implementation studies.

## 1 Background

National statistics show that, at the end of 2024, China had 310.31 million people aged 60 years or older, accounting for 22.0% of the national population (1). Among them, 46.54 million older adults require assistance with daily activities due to age-related declines in physical function and limitations or loss of self-care ability (2). With the accelerated progression of population aging in China (3), promoting healthy aging has become a national priority. In recent years, the Chinese government has elevated active responses to population aging to a national strategy. The Healthy Aging Plan for the 14th Five-Year Plan Period emphasizes maintaining physical function and preserving older adults’ ability to live independently as key objectives of healthy aging (4).

In Japan, support for independence is a foundational principle of the long-term care insurance system and is intended to enable older adults who require care to continue living in accordance with their own wishes (5). Previous studies have shown that care programs guided by independence-supportive principles can improve older adults’ physical functioning, alleviate depressive symptoms, and enhance quality of life. Such programs may also reduce caregiver burden, long-term care costs, and financial strain on families. In this study, independence-supportive care is used as the principal term for an approach in which nursing assistants enable older adults to use their retained abilities and participate in daily activities with the least assistance necessary, while respecting safety and individual preferences. Independence-supportive care overlaps with reablement, restorative care, and function-focused care in emphasizing function, participation (6–8).

In Chinese nursing homes, nursing assistants are distinct from registered nurses and provide most hands-on daily care to older adults. China’s national occupational skills standard specifies no minimum level of formal education for entry into this occupation. Individuals with primary school education, including those entering aged care from other occupations, may qualify after completing the required training or gaining relevant work experience and passing theoretical and practical skills assessments. Their duties extend beyond assistance with basic activities of daily living. Depending on their skill level, they may also provide rehabilitation and functional support, psychological support, health observation, and communication with residents and families. The workforce therefore includes individuals with varied educational and occupational backgrounds, which may result in differences in professional preparation and understanding of independence-supportive care. Because nursing assistants make day-to-day decisions about whether to encourage residents to perform an activity, provide partial assistance, or take over the task, they play a central role in supporting residents’ remaining abilities. Throughout this manuscript, the term nursing assistant refers specifically to this occupational group and does not include registered nurses, rehabilitation therapists, social workers, facility managers, or other professionals.

The application of independence-supportive care in China remains at an exploratory stage. Existing qualitative research (9) has identified factors affecting its implementation at the levels of intervention characteristics, organizational context, and care recipients, with participants including managers, nursing assistants, and older residents. Although these studies provide an initial understanding of the conditions required for implementation, limited attention has been given to how frontline nursing assistants make care decisions in everyday practice and how these decisions are shaped. Consequently, it remains unclear under what conditions nursing assistants are willing to initiate and sustain independence-supportive care.

The Capability, Opportunity, Motivation–Behavior (COM-B) model proposes that behavior results from the interaction of capability, opportunity, and motivation (10). Capability refers to the knowledge, judgment, and practical skills required to perform a target behavior. Opportunity encompasses the environmental and social conditions that enable or constrain the behavior. Motivation includes the intentions, beliefs, and psychological processes that initiate and sustain it. By considering both individual characteristics and organizational context, the COM-B model provides an appropriate theoretical framework for systematically examining factors that influence nursing assistants’ implementation of independence-supportive care.

In this study, the target behavior was defined as nursing assistants’ actions during daily care to support older residents with functional limitations in using their retained abilities to participate in activities within their capabilities. A descriptive qualitative design was therefore used to explore nursing assistants’ experiences of providing independence-supportive care and the factors influencing their practices. The findings may inform improvements in training, organizational support, and the implementation of independence-supportive care in nursing homes.

## 2 Methods

### 2.1 Study design and reporting

This descriptive qualitative study examined nursing assistants’ experiences and decisions when providing independence-supportive care during everyday work. Reporting was guided by the Consolidated Criteria for Reporting Qualitative Research (COREQ) (11).

### 2.2 Study participants

A two-stage purposive sampling strategy was used. Five nursing homes in Hunan Province were purposively selected to provide variation in geographic location, ownership, and service model. The facilities included public and private nursing homes with different service orientations and fee levels. Eligible nursing assistants were subsequently recruited from these facilities. The characteristics of the participating facilities are presented in Table 1. At the participant level, nursing assistants were eligible if they (1) worked in one of the participating nursing homes, (2) had at least 1 year of nursing home work experience, (3) held a nursing assistant certificate, and (4) agreed voluntarily to participate. Individuals who were unable to clearly describe their experiences were excluded.

**Table 1:** Sociodemographic and professional characteristics of participants (*n* = 12).

Characteristic	Category	*n*	%
Sex	Male	2	16.7
	Female	10	83.3
Age, years	≤30	3	25.0
	31–40	1	8.3
	>40	8	66.7
Work experience, years	≤5	4	33.3
	6–10	3	25.0
	>10	5	41.7
Education	Primary school	2	16.6
	Middle/high school	5	41.7
	College or above	5	41.7
Facility type	Public nursing home	3	25.0
	Other nursing home	6	50.0
	High-end nursing home	3	25.0

Public nursing homes were publicly operated; high-end nursing homes were higher-fee facilities; and other nursing homes were non-public facilities not classified as high-end. Participant counts by facility are withheld to reduce re-identification risk. Bed capacity and rehabilitation-resource data were not collected consistently.

After approval was obtained from the administrators of the participating nursing homes, the administrators acted solely as institutional gatekeepers by distributing recruitment information to nursing assistants who might meet the eligibility criteria. The recruitment materials included the researcher’s telephone number. Nursing assistants interested in participating contacted the researcher directly and were subsequently screened for eligibility and informed about the study purpose and procedures. Participation was voluntary. Facility administrators were not informed about which nursing assistants contacted the research team and were not involved in eligibility screening, decisions about participation, informed consent, or the interviews.

Because recruitment information was distributed by facility administrators and interested nursing assistants contacted the researcher directly, the research team did not have access to complete staff lists. The total number of nursing assistants employed across the five facilities, the number who met the eligibility criteria, the number who received the recruitment information, and the number who declined or did not respond were not systematically recorded. A participation rate could therefore not be calculated.

A total of 15 nursing assistants were recruited. No participant withdrew after enrollment. Three participated in pilot interviews conducted solely to refine the interview guide, and their interview data were not included in the final analysis. The final analytic sample comprised 12 nursing assistants who completed the main-study interviews. Participants were assigned anonymized codes N1–N12, and all quotation identifiers were checked against the final analytic dataset. Both face-to-face and telephone interviews were conducted. Interview mode was not retained as an analytic characteristic, and a reliable numerical breakdown by interview mode was therefore unavailable.

After each main-study interview, two researchers compared the newly generated codes and provisional themes with the developing codebook. A third researcher participated in the final assessment of data sufficiency. Recruitment ended when successive interviews generated no substantively new codes, relationships among codes, or changes to the provisional thematic structure (12). Participants’ nursing home work experience ranged from 1 to 17 years, with a median of 8 years.

The study was approved by the Biomedical Research Ethics Committee of Hunan Normal University (approval no. 2025-840). The study involved human participants, and all participants provided written informed consent before data collection.

### 2.3 Interview guide

We systematically reviewed the literature on independence-supportive care and identified potential factors that could influence implementation by nursing assistants. The research team discussed and revised a preliminary interview guide. Pilot interviews were then conducted with eligible participants. Their feedback was used to improve clarity and remove ambiguity. The final questions were:

1. Have you heard of independence support or assistance for independence? How do you understand this concept?
2. Do you encourage functionally dependent older adults to do tasks that remain within their ability, such as eating or dressing by themselves?
3. What benefits may older adults gain from doing tasks within their ability?
4. Do you think encouraging older adults to do these tasks involves risks? What risks have you encountered?
5. What difficulties does this approach create for nursing assistants? Please give examples.
6. What are the main care needs of the older adults you currently support?
7. Do you support independence-supportive care? Why?
8. Under what circumstances do you encourage an older adult to do tasks within their ability?

### 2.4 Interview procedures

Before each interview, the interviewer introduced herself by name and informed participants of her affiliation with Hunan Normal University, her role as a master’s nursing student and interviewer, and that the study was conducted under the supervision of her academic supervisor. She then explained the study purpose, procedures, voluntary nature of participation, confidentiality protections, expected interview duration, and audio-recording arrangements. All participants provided written informed consent before the interview. Face-to-face interviews were conducted in a quiet, private room within the participating nursing homes, with only the participant and interviewer present. Participants interviewed by telephone had received the study information, signed and returned the informed consent form, and agreed on a suitable interview time before the call. They were asked to take the call alone in a private setting where they would not be interrupted or overheard. At the beginning of each telephone interview, participants confirmed that no other person was present and that the setting provided adequate privacy. The interviewer then reiterated the study purpose and procedures and verbally reconfirmed the participant’s willingness to participate and consent to audio recording before beginning the interview.

During the interviews, the researcher followed the interview guide while encouraging participants to elaborate on their personal perspectives, experiences, and feelings. Each interview lasted approximately 20–30 min. No repeat interviews were conducted.

### 2.5 Interview materials

The researcher served as the interviewer and independently conducted all interviews. Before each interview, an audio recorder was prepared to ensure complete recording of the interview data. A notebook and writing materials were also used to document emerging themes, capture potential areas for further exploration, and record participants’ nonverbal information during the interviews.

### 2.6 Researcher characteristics

The first author, a female graduate student who held a bachelor’s degree in nursing and an intermediate-level professional title and was pursuing a master’s degree in nursing, conducted all interviews. She also had previous frontline experience in long-term care. She had no personal, family, or pre-existing caregiving relationships with the participants. Her previous experience helped her understand care-specific terminology and the organizational context described by participants, which facilitated rapport and follow-up probing. However, this background may also have sensitized her to issues such as workload, safety, and organizational constraints and could have influenced which aspects of participants’ accounts she explored or considered salient. Participants may also have perceived her as familiar with long-term care practice, which could have encouraged professionally favorable or socially desirable accounts of their work. To manage these potential influences, the interviewer used open-ended questions and neutral probes, asked participants to describe concrete experiences in their own words, and avoided expressing approval or disapproval of their responses. Within 24 h of each interview, she completed a reflexive journal to identify possible leading questions, overlooked information, and ways in which her professional background or interaction with the participant might have influenced the interview. These reflections informed subsequent interviews. During analysis, two graduate researchers independently coded the original Chinese transcripts, and disagreements and alternative interpretations were discussed with a third researcher until consensus was reached. The COM-B model was applied only after the inductive themes had been developed and therefore did not predetermine the initial coding or theme-generation process.

### 2.7 Data analysis

NVivo 15.0 was used to organize and analyze the data. The data were analyzed using a practical, codebook-oriented thematic analysis approach (13). Before coding began, the researchers read the original transcripts repeatedly to familiarize themselves with the data and to identify and mark segments relevant to the research question. Initial codes were then generated, compared for similarities and relationships, and grouped into subthemes. To ensure that the themes accurately reflected the original data, the researchers revisited the transcripts, reviewed the subthemes, and examined the relationships among them. Two graduate researchers independently completed the initial reading and coding. Any disagreements were discussed with a third researcher until consensus was reached, resulting in a preliminary coding framework. The COM-B model was used only to interpret the themes after they had been developed; it did not inform the initial coding or theme-generation process.

### 2.8 Quality control

#### 2.8.1 Before interviews

The researcher completed online training in qualitative research methods and interviewing techniques. Communication was established with administrators of long-term care facilities. Rapport was established during pre-interview communication and at the beginning of each interview. Written informed consent was obtained from all participants.

#### 2.8.2 During interviews

Interviews were conducted in quiet, private, and interruption-free environments. Participants were informed that the interviews would be audio-recorded. After obtaining consent, the entire interview was recorded, and key points were documented. Throughout the interviews, the researcher used neutral follow-up probes and avoided expressing approval or disapproval of participants’ responses. Within 24 h after each interview, the researcher completed reflective journals to review whether any leading questions had occurred or whether important information had been overlooked, which informed subsequent interviews.

#### 2.8.3 During data management and analysis

Interview transcripts were completed within 24 h after each interview to minimize the loss of important details. Neither the full interview transcripts nor the preliminary findings were returned to participants for comment, correction, or validation. Participants were contacted only when clarification of an unclear statement was required.

### 2.9 Transcription, translation, and data management

Interviews were conducted in Chinese, and transcribed verbatim in Chinese within 24 h. Coding and theme development used the original Chinese transcripts. ChatGPT was used only to assist with translating selected participant quotations into English and back-translating them into Chinese. No independent human translator was used. Members of the research team compared each back-translation with the corresponding original Chinese quotation and resolved discrepancies before reporting it. ChatGPT was not used for coding, theme development, or interpretation.

Audio files, transcripts, and analytic files were stored. Identifiers were removed from transcripts, and participants were represented by analytic codes N1–N12.

## 3 Results

Five themes were identified: (1) knowledge and skills related to independence-supportive care remained unstandardized; (2) residents’ functional status, willingness, and expectations of care affected their participation; (3) poor integration between assessment and care planning prevented retained abilities from becoming daily care goals; (4) limited staffing, time, and assistive devices led nursing assistants to take over residents’ tasks; and (5) safety risks and accountability pressures promoted risk-averse care. After inductive theme development, the five themes and their subthemes were mapped to the COM-B domains (Figure 1 and Table 2).

**Figure 1:**
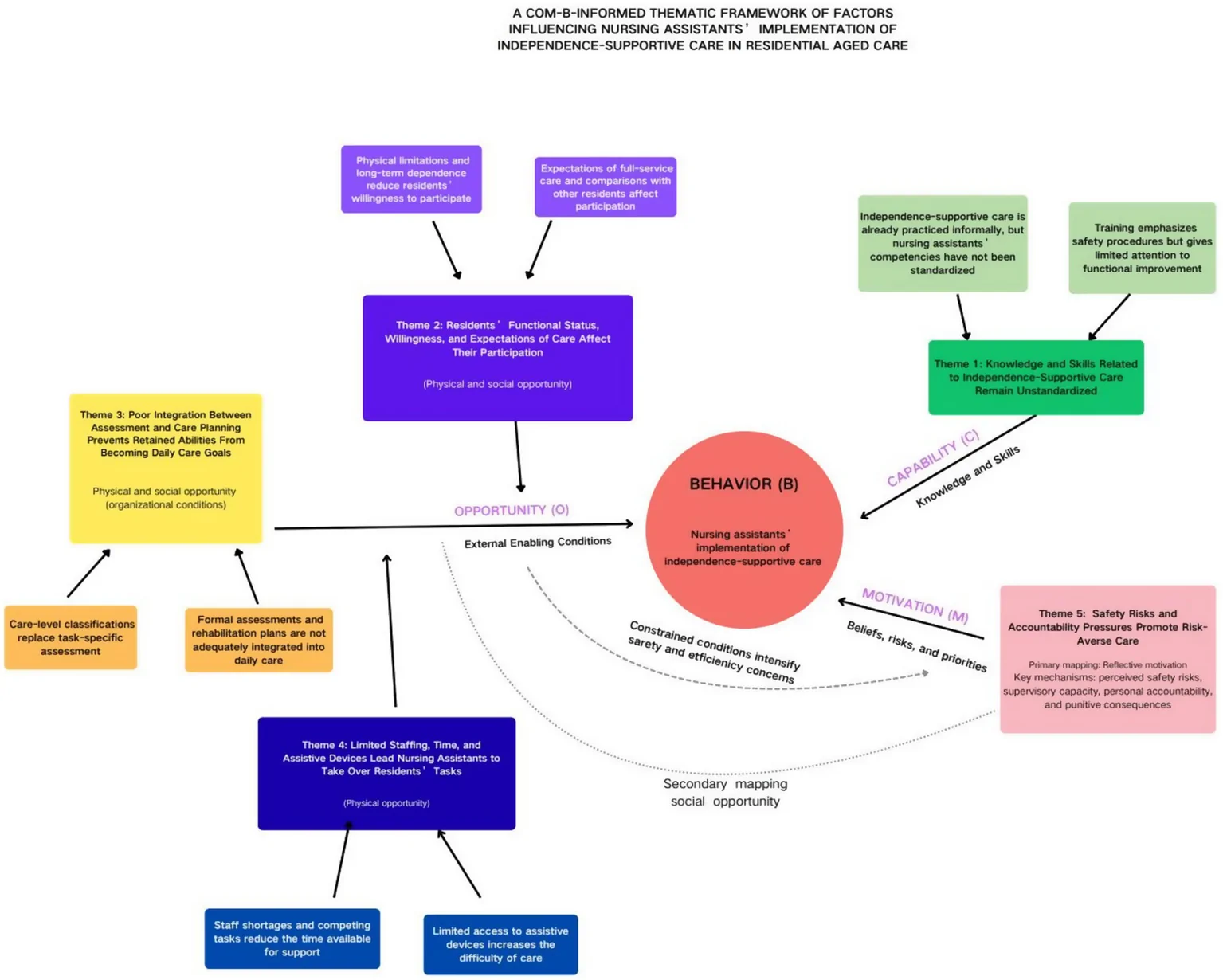
*Post hoc* mapping of the inductively developed themes to the COM-B domains.

**Table 2:** Mapping of themes and subthemes to COM-B domains, implementation role, and representative quotations.

Theme or subtheme	COM-B domain	Role	Representative quotation
Theme 1.1 Informal practice with unstandardized competencies	Capability	Mixed	“I have not heard those formal terms before, but I know that moving around is good for older adults.” (N4)
Theme 1.2 Safety-focused training with limited attention to function	Capability	Barrier	“Our examination covered fall prevention, pressure injury prevention, wheelchair transfers, and stretcher transfers.” (N2)
Theme 2.1 Physical limitations and long-term dependence	Physical and social opportunity	Barrier	“When we ask them to walk regularly, some say it is too tiring and refuse.” (N2)
Theme 2.2 Expectations of full-service care and comparisons	Social opportunity	Barrier	“Family members usually want you to do more so that the resident can do less.” (N12)
Theme 3.1 Care-level classifications replace task-specific assessment	Physical and social opportunity	Barrier	“We work according to the level listed on the assessment form.” (N10)
Theme 3.2 Assessment and rehabilitation plans are poorly integrated into daily care	Physical and social opportunity	Barrier; collaboration is an enabler	“We are responsible for residents’ daily care, but we are not fully involved in discussions about their rehabilitation plans.” (N8)
Theme 4.1 Staff shortages and competing tasks	Physical opportunity	Barrier	“We are short-staffed, and one person has only so much energy.” (N9)
Theme 4.2 Limited access to assistive devices	Physical opportunity	Barrier	“Our facility does not provide these devices, but we recommend that families purchase them.” (N12)
Theme 5 Safety risks and accountability pressures	Reflective motivation; secondary social opportunity	Barrier; manageable risk and supervision are enablers	“If they are accidentally injured, we are the ones who have to take responsibility.” (N9)

### 3.1 Theme 1: knowledge and skills related to Independence-supportive care remain unstandardized

#### 3.1.1 Independence-supportive care is already practiced informally, but nursing assistants’ competencies have not been standardized

Most nursing assistants had not heard of the concept of independence-supportive care, indicating that it remains unfamiliar among nursing assistants in long-term care facilities. Nursing assistants with formal education in eldercare-related fields were also unfamiliar with the term itself, but they had encountered similar principles and skills during their education. They understood the importance of helping older residents maintain or improve their existing functional abilities.

*“I’ve never heard of independence-supportive care. Does it mean volunteering?”* (N2)

*“I haven’t heard those formal terms before, but I know that moving around is good for older adults. If you always leave them in bed, they become less and less willing to move.”* (N4)

*“When I was in school, I regularly took part in skills competitions. We were always taught to encourage older adults to do as much as possible by themselves. That way, they could maintain their current physical abilities or gradually improve.”* (N3)

Although most nursing assistants lacked systematic knowledge of independence-supportive care, some assessed residents’ ability to eat, brush their teeth, or move based on personal experience, discussions with coworkers, and informal trials during daily care.

*“For residents with disabilities, I first let them try using a spoon by themselves. If they really can’t manage it, then I feed them. I also encourage them to move their limbs and joints.”* (N6)

*“We talk with one another about the details of each resident’s care. We learn about their habits and preferences and gradually figure out the best way to care for them.”* (N8)

#### 3.1.2 Training emphasizes safety procedures but gives limited attention to functional improvement

Training provided by vocational schools and most long-term care facilities focused primarily on fall prevention, infection control, first aid, repositioning, transfers, and basic assistance with activities of daily living. Training in functional improvement was limited.

*“Our examination covered fall prevention, pressure injury prevention, wheelchair transfers, and stretcher transfers. We were expected to know most of these procedures.”* (N2)

*“Our department provides training every month and every year. There are so many procedures, such as wheelchair transfers and the Heimlich maneuver. Most of them are basic care procedures and first-aid techniques. There are so many that I can’t even remember all their names.”* (N5)

A small number of participants from better-resourced facilities reported that, although their facilities did not explicitly use the term *independence-supportive care*, they conveyed similar principles through reablement and rehabilitation training and provided instruction in related skills.

*“Our facility has discussed a similar concept called reablement. It means helping older residents demonstrate what they can still do at this stage of life and giving them a sense of achievement. It is very similar to independence-supportive care.”* (N1)

*“What you described as independence-supportive care means allowing older residents to dress and stand by themselves. The rehabilitation services we provide include dynamic balance, standing balance, walking, dressing and undressing, and eating. These are covered in our facility’s training.”*(N12)

### 3.2 Theme 2: residents’ functional status, willingness, and expectations of care affect their participation

#### 3.2.1 Physical limitations and long-term dependence reduce residents’ willingness to participate

Nursing assistants described residents’ physical and cognitive conditions as influencing both their ability and willingness to participate in daily activities. Some residents had difficulty eating, dressing, or walking independently because of muscle weakness, prolonged bed rest, or cognitive impairment. Fatigue, pain, dizziness, and fear of falling during an activity further discouraged them from trying.

Other residents had become accustomed to staying in bed or relying on nursing assistants after receiving extensive assistance over a long period. They lacked confidence in resuming independent activities and believed that exercise would be of little benefit at their age.

*“The residents who come here are usually physically weak. When we ask them to walk regularly, some say it is too tiring and refuse. We can’t force them.”* (N2)

*“Sometimes the residents themselves are the main barrier. They think they’re too old for these activities to make any difference, so they don’t want to do them.”* (N3)

*“There was an older woman who wasn’t used to lying in bed and insisted on sitting in a wheelchair. Later, she fractured her leg and couldn’t move or sit in the wheelchair. Now she has become used to staying in bed. When we ask her to sit in the wheelchair, she refuses and says she feels dizzy when she sits up. She has become so used to lying down that she no longer wants to get out of bed.”* (N11)

When residents were reluctant to participate, nursing assistants generally used explanations, encouragement, and praise to persuade them to try. If a resident clearly refused and could not continue, nursing assistants reduced the intensity of the activity, provided additional assistance, or respected the resident’s choice.

*“Some residents simply don’t want to do it because they think it’s too much trouble. I used to spend a lot of time encouraging one resident to walk more instead of always sitting in a wheelchair. But the more I talked about it, the more annoyed he became, so eventually I stopped.”* (N5)

*“If a resident doesn’t want to move, there isn’t much we can do. We can keep talking to them, but that’s about it. We have one older man who understands everything clearly but can’t express himself well. He just doesn’t want to move. When we remind him, he moves a little. If we don’t remind him, he does nothing. We see all kinds of residents here.”* (N9)

Residents’ participation in independent activities was therefore not determined by willingness alone. It reflected the combined influence of physical ability, cognitive status, previous experiences during activities, and patterns of dependence established over time.

#### 3.2.2 Expectations of full-service care and comparisons with other residents affect participation

Some residents and family members viewed payment for residential care as the purchase of a full-service arrangement in which nursing assistants were expected to perform all daily activities, including feeding, dressing, and washing. Within this understanding of paid care, encouraging residents to complete activities within their abilities could be interpreted as a failure to provide adequate service.

Even when residents retained some ability to perform these activities, they or their family members sometimes requested more assistance on the grounds that they had paid for the service. Some family members judged care quality according to how many tasks nursing assistants completed and expected staff to “do more so that the resident could do less.” This placed nursing assistants in the difficult position of balancing functional maintenance with family expectations.

*“When you explain independence-supportive care to family members, they may say it isn’t necessary. They believe they sent the resident here so that everything would be taken care of.”* (N1)

*“For example, if this were my father and I couldn’t care for him properly at home, I would pay to have him cared for here. Ninety percent of families are satisfied with the care we provide, but a few still think it isn’t enough, even when the resident is being well cared for.”* (N2)

*“Family members usually want you to do more so that the resident can do less. I think that is probably the biggest issue.”* (N12)

Residents also compared the care they received with that provided to others in the same facility. When they saw other residents being fed or receiving full assistance with daily activities, some residents who remained partly independent felt that they were being treated differently and requested the same level of assistance.

*“Some residents simply don’t want to make the effort. They think, ‘I’ve already paid, so why should I have to do these things? The nursing assistants should do them for me.’ Then they refuse to cooperate.”* (N9)

*“Some residents see other people receiving help and start relying on us too. When we tell them to do something themselves, some will try, but others refuse even after we talk to them. They say, ‘I paid for this.’ Some see nursing assistants feeding other residents and then want to be fed too.”* (N11)

### 3.3 Theme 3: poor integration between assessment and care planning prevents retained abilities from becoming daily care goals

#### 3.3.1 Care-level classifications replace task-specific assessment

Care-level classifications were used not only to allocate institutional resources but also to determine the daily tasks nursing assistants were expected to perform. Most nursing assistants provided care according to the assigned level and associated admission to a long-term care facility or severe disability with complete dependence. Within this model of care, residents’ retained abilities could easily go unnoticed.

*“If the residents here could take care of themselves, their families wouldn’t have sent them here.”* (N5)

*“We work according to the level listed on the assessment form. For example, if the physician and nurse classify a resident as requiring Level 1 care, we provide care according to that classification.”* (N10)

*“The residents I care for have disabilities and have been bedridden for a long time. Our staff have to do everything for them.”* (N6)

#### 3.3.2 Formal assessments and rehabilitation plans are not adequately integrated into daily care

In most facilities, physicians, nurses, rehabilitation therapists, or designated assessors were responsible for evaluating residents’ abilities and developing rehabilitation plans. Nursing assistants were primarily responsible for providing daily care and carrying out prescribed rehabilitation activities.

Nursing assistants spent the most time with residents and were able to observe changes in physical function and cooperation during eating, dressing, toileting, and mobility. However, these observations were usually communicated informally or during shift handovers and were rarely incorporated systematically into formal assessments or rehabilitation plans.

*“Our facility employs professional assessors, and physicians and nurses are responsible for residents’ rehabilitation. We do what they tell us to do. At most, we report whether a resident is cooperating.”* (N8)

*“I have an associate degree in eldercare services and obtained an assessor qualification last year, but assessments are still conducted by the eldercare services department. The physicians don’t listen to us when it comes to rehabilitation.”* (N10)

*“We assess residents’ self-care abilities based on our own experience. If we observe that a resident can complete an activity, we pass that information on to the next shift so they know the resident can do it independently.”* (N12)

Some participants reported that they had little involvement in discussions about rehabilitation goals, activity selection, or the appropriate level of assistance. They generally followed plans developed by other professionals. Because formal assessments and daily care were handled by different occupational groups, interim rehabilitation goals were not always translated promptly into requirements for eating, dressing, washing, toileting, and mobility. Likewise, subtle functional changes observed by nursing assistants might not reach those responsible for revising the plan.

*“We are responsible for residents’ daily care, but we are not fully involved in discussions about their rehabilitation plans. The physicians are responsible for that.”* (N8)

*“We didn’t participate in that program at all.”* (N6)

*“When we notice changes in a resident’s physical condition, we communicate with the rehabilitation therapist. However, the therapist mainly discusses the rehabilitation plan with the family.”* (N1)

A small number of facilities had developed clearer collaborative arrangements. Rehabilitation therapists conducted professional assessments and developed plans, while nursing assistants helped residents practice during daily activities. Regular communication allowed nursing assistants to report functional changes and extend short-term rehabilitation exercises into everyday care.

*“The rehabilitation therapist assesses each resident and develops an individualized rehabilitation plan. Our role is to support the exercises during daily activities and help residents complete tasks they were previously unable to perform.”* (N7)

*“We use an integrated model involving social work, rehabilitation, and care. The three groups meet regularly to discuss each resident’s situation and plan follow-up.”* (N3)

### 3.4 Theme 4: limited staffing, time, and assistive devices Lead nursing assistants to take over residents’ tasks

#### 3.4.1 Staff shortages and competing tasks reduce the time available for support

Providing independence-supportive care required nursing assistants to explain each step repeatedly, remain nearby to ensure safety, stabilize assistive devices, and complete the necessary cleanup afterward. Participants reported that staff shortages and competing care demands often prompted them to take over residents’ tasks because doing so allowed them to complete their work more quickly.

*“We can finish bathing a resident very quickly if we do it ourselves. When residents bathe themselves, they move slowly, so it takes much more of our time and energy.”* (N4)

*“We are short-staffed, and one person has only so much energy. Caring for seven or eight residents is already exhausting.”* (N9)

*“We care for a lot of residents. I’m responsible for six. It isn’t realistic to help them practice walking every day. At most, we do it once a week and rotate among residents.”* (N10)

#### 3.4.2 Limited access to assistive devices increases the difficulty of care

Facilities provided a limited range of assistive devices because of cost considerations. When suitable equipment was unavailable, nursing assistants had to manually lift or support residents with disabilities during transfers. Nursing assistants perceived inadequate access to assistive devices as increasing their physical burden and limiting residents’ opportunities to participate in transfers and other activities.

*“Our facility doesn’t provide these devices, but we recommend that families purchase them. If the facility had to provide everything, the cost would be very high.”* (N12)

*“We have some assistive devices and provide the basic ones. For residents with special needs, we may offer a temporary loan or rental service. If we don’t have what they need, the family has to purchase it.”* (N9)

*“If we can’t move a resident and the resident’s son is there, we ask him to help. Sometimes coworkers help one another.”* (N6)

### 3.5 Theme 5: safety risks and accountability pressures promote risk-averse care

When residents ate, dressed, bathed, or walked independently, nursing assistants considered the risks of falls, choking, and accidental injury. Most did not oppose independent activity outright. Instead, they decided whether to provide an opportunity based on the difficulty of the activity, the resident’s physical condition, and whether close supervision was possible.

Some nursing assistants encouraged residents to try when the activity remained within a range they could supervise and control. When they considered the risk beyond their ability to assess or manage, they were more likely to restrict the activity or provide direct assistance.

*“If the resident is within my sight and is practicing something they can manage independently, I think that is a good thing.”* (N8)

*“I’m willing to let residents be active as long as it is within my ability to manage. If the activity is too difficult, I put safety first.”* (N11)

*“Our facility emphasizes safety above everything else. If a resident can use the toilet independently, I support that. But if there is a high risk of falling, I don’t.”* (N1)

Nursing assistants’ concerns extended beyond possible harm to residents. They also worried about complaints from family members, criticism from facility managers, and financial penalties following an adverse event. Participants reported becoming more cautious when they believed that they would bear personal responsibility for an activity-related incident. They described restricting independent activity and taking over tasks as safer and more controllable responses.

*“We do want residents to move more. The more they move, the less likely their abilities are to deteriorate. But we worry that if something goes wrong, money will be deducted from our pay.”* (N6)

*“I still don’t dare let residents move around by themselves. If they are accidentally injured, we are the ones who have to take responsibility.”* (N9)

*“I prefer caring for residents who are bedridden because at least there is no risk of them falling. With bedridden residents, the main tasks are preventing pressure injuries, helping with meals, and providing personal hygiene.”* (N12)

Nursing assistants’ risk-averse practices did not necessarily indicate rejection of independence-supportive care. Rather, these practices reflected decisions made in the absence of clear risk classifications, operational boundaries, and shared accountability mechanisms.

## 4 Discussion

The findings were interpreted using the Capability, Opportunity, Motivation–Behavior (COM-B) model. Nursing assistants’ implementation of independence-supportive care was shaped by the interaction of capability, opportunity, and motivation.

Capability included the knowledge and skills needed to identify residents’ retained functions, assess task-specific abilities, and provide graded assistance. Opportunity included adequate staffing and time, suitable assistive devices, interprofessional collaboration, and organizational support. Motivation included recognition of the value of independence-supportive care, as well as concerns about safety, personal accountability, and completing required tasks during a shift. These concerns could lead nursing assistants to perform activities for residents even when they supported the general principle underlying independence-supportive care.

### 4.1 Standardizing training to strengthen nursing assistants’ capability

Training in most long-term care facilities continued to focus on safety and the completion of basic care tasks, with limited attention to specific methods of independence-supportive care. Nursing assistants therefore had considerable room to strengthen their competency in this area.

Although most participants were unfamiliar with the professional term *independence-supportive care*, some could assess residents’ ability to perform specific activities based on daily observations and prior experience. They supported residents by encouraging them to try and by providing partial assistance when needed. Previous studies have similarly found that some long-term care facilities already use practices resembling independence-supportive care (14, 15). However, these practices generally arise from nursing assistants’ accumulated experience and lack consistent criteria and operating procedures.

Hartig et al. (16) compared functional assessments completed by nursing assistants and clinical nurse specialists in long-term care facilities. After nursing assistants received training in the use of standardized instruments, their assessments showed a high level of agreement with those of the clinical nurse specialists. This finding suggests that nursing assistants are capable of contributing to functional assessment when they are given practical standardized tools and appropriate training.

The findings suggest that facilities may need to supplement overall care-level classifications with assessments of residents’ task-specific abilities. Future intervention studies should examine whether this approach improves the appropriateness and consistency of assistance. It should also reflect residents’ actual ability to eat, dress, groom, use the toilet, and move. Training should focus on these common activities and teach nursing assistants how to identify retained abilities, break tasks into manageable steps, select an appropriate level of assistance, recognize risks, and evaluate outcomes.

Scenario-based simulations and workplace competency assessments could be considered as possible methods for evaluating nursing assistants’ skills after training. However, the effectiveness of these methods in standardizing independence-supportive care was not examined in the present study.

### 4.2 Improving working conditions and collaborative support to increase opportunities for implementation

Within the COM-B model, opportunity refers to the external conditions that make independence-supportive care possible. These conditions include staffing, protected time, and assistive devices, as well as cooperation from residents and families, organizational support, and interprofessional collaboration.

Staff shortages, competing tasks, and limited time reduced nursing assistants’ ability to support residents’ independent activities. This finding is consistent with that of Liao et al. (17), who reported that workload, organizational conditions, and external support directly affected nursing assistants’ practices.

Assistive devices are also essential to independence-supportive care. Appropriate equipment can reduce nursing assistants’ physical burden and create safer opportunities for residents to participate in daily activities. Previous studies have shown that assistive technologies can support older adults in performing activities of daily living and may reduce the workload of paid caregivers (18, 19).

In addition to material resources, the cooperation of residents and families affected whether independence-supportive care could be implemented. In this study, residents and family members sometimes interpreted encouragement of independent activity as inadequate service. Park et al. (20) similarly found that residents’ expectations of dependence and differences between staff and family expectations could interfere with residents’ participation in self-care. Independence-supportive care therefore depends not only on individual nursing assistants but also on working conditions and the expectations held by residents and their families.

A small number of cases in the present study showed that independence-supportive care was more likely to continue when social workers, rehabilitation therapists, and nursing assistants communicated regularly and nursing assistants reinforced rehabilitation goals during everyday care. Previous studies have also indicated that nursing assistants’ participation in interdisciplinary collaboration helps extend short-term rehabilitation goals into residents’ daily routines (21, 22).

The findings identify staffing, workflow organization, assistive devices, communication with families, and interprofessional collaboration as potential targets for supporting implementation. Further research is needed to determine whether changes in these areas lead to sustained changes in care practice. Facilities could document residents’ current abilities, required levels of assistance, and relevant risks in care plans and shift-handover records, giving nursing assistants a clear basis for daily practice.

During admission discussions and care planning, facilities should explain to residents and families that independence-supportive care does not mean reducing services. Rather, it involves providing the appropriate level of assistance so that residents can maintain their existing abilities within safe limits. Clear communication may improve residents’ and families’ understanding of the goals of care, although this effect was not evaluated in the present study.

### 4.3 Strengthening risk management and service quality evaluation to support motivation

Most participants believed that independence-supportive care might help residents maintain or improve their physical functioning and could potentially reduce future care needs. These statements reflected participants’ perceived benefits rather than outcomes measured in the present study. Such perceived benefits appeared to support their willingness to implement this approach. However, participants also described safety concerns, personal accountability, financial penalties, and pressure to complete work efficiently as factors that could weaken this willingness.

Previous studies have similarly found that complaints from family members may lead to formal meetings or wage deductions for nursing assistants (23–25). To avoid these consequences, nursing assistants may restrict residents’ activities or take over tasks to reduce uncertainty and risk.

Participants’ accounts suggest that emphasizing the potential benefits of independence-supportive care alone may be insufficient to change reported practice. Based on these accounts, activity-specific risk guidance, graded assistance criteria, and documentation in individual care plans may be considered as possible organizational responses.

Facilities could consider conducting an interdisciplinary assessment at admission and informing families of the goals, procedures, and potential risks of the care plan. One possible organizational response would be to review adverse events through an interdisciplinary process rather than automatically attributing responsibility to the frontline nursing assistant. The feasibility and effects of such a shared-accountability approach require further evaluation.

Quality evaluation could extend beyond the occurrence of adverse safety events to assess whether residents’ abilities were adequately evaluated, whether the level of assistance was appropriate, and whether existing functions were used and maintained.

Overall, nursing assistants’ reported tendency to take over tasks should not be interpreted solely as a lack of responsibility or rejection of independence-supportive care. Their accounts suggest that this tendency may represent a response to limited capability, constrained working conditions, and unclear accountability.

The COM-B model helps explain how these factors are connected. Limited knowledge and skills reduce nursing assistants’ confidence in providing independence-supportive care. Inadequate time, staffing, and professional support make implementation more difficult. These conditions, in turn, intensify concerns about safety and efficiency. The findings identify nursing assistants’ capability, everyday working conditions, professional support, and accountability arrangements as possible intervention targets. They also suggest that approaches focused only on changing nursing assistants’ attitudes may not address the organizational constraints described by participants. Implementation and intervention studies are needed to determine whether combined changes in training, staffing, professional support, and accountability lead to observable improvements in care practice or resident outcomes.

## 5 Limitations

Participants were purposively sampled from nursing homes in Hunan Province. Although maximum variation was sought, regions differ in the development of aged-care services, facility models, staffing, and adoption of independence-supportive care. The transferability of the findings is therefore limited. Future studies should include participants from different regions and types of facilities. They should also examine more closely how acceptance of the concept is translated into care behavior. Recruitment figures were not systematically recorded, so a participation rate could not be calculated. As participation was voluntary, the possibility of self-selection should be considered when interpreting the findings. The findings relied on nursing assistants’ self-reported practices and were not corroborated through direct observation. Questions about perceived benefits and support for independence-supportive care may have encouraged socially desirable responses despite the use of neutral probes and reflexive journaling. Recruitment figures were not systematically recorded because facility administrators distributed the recruitment information and interested nursing assistants contacted the researcher directly. Consequently, administrator-mediated distribution of recruitment information may also have influenced who received or felt able to respond to the invitation. These factors should be considered when assessing the transferability of the findings. Finally, AI-assisted translation and back-translation may have altered nuances in participants’ original Chinese expressions, even though the research team compared the back-translated wording with the original transcripts. The absence of consistently collected facility-level size and rehabilitation-resource data further limits contextual comparison and transferability.

## Data Availability

The original contributions presented in the study are included in the article/Supplementary material, further inquiries can be directed to the corresponding author.
