# Supplementary material for: Behavioral choices and enabling conditions for implementing independence-supportive care among nursing assistants in residential aged care facilities in Hunan Province, China: a qualitative study
Source: Front Public Health. 2026 Oct 6;14:1972560. doi: 10.3389/fpubh.2026.1972560 (PMC13682637; doi:10.3389/fpubh.2026.1972560)
Supplement: Supplementary file 1 [file Supplementary_file-_1.docx]

**COREQ 32-Item Checklist**

**Consolidated Criteria for Reporting Qualitative Research: Interviews and Focus Groups**

Behavioral choices and enabling conditions for implementing independence-supportive care among nursing assistants in residential aged care facilities in Hunan Province, China: A qualitative study

This checklist reflects the revised manuscript. “Reported” means the manuscript explicitly addresses the item, including when a procedure was not undertaken. “Partially reported” identifies information that remains incomplete. “Unavailable” identifies recruitment counts that were not systematically recorded.

| **No.** | **COREQ item** | **Reporting requirement** | **Status** | **Manuscript location** | **Evidence in the final manuscript** |
| --- | --- | --- | --- | --- | --- |
| 1 | **Interviewer/facilitator** | Which author(s) conducted the interview or focus group? | reported | Methods, 1.6 | The first author conducted all interviews. |
| 2 | **Credentials** | What were the researcher's credentials? | reported | Methods, 1.6 | The first author, a female graduate student who held a bachelor’s degree in nursing and an intermediate-level professional title and was pursuing a master’s degree in nursing, conducted all interviews. She also had previous frontline experience in long-term care. |
| 3 | **Occupation** | What was the researcher's occupation at the time of the study? | Reported | Methods, 1.6 | The interviewer was a graduate nursing student with previous frontline long-term-care experience. |
| 4 | **Gender** | Was the researcher male or female? | Reported | Methods, 1.6 | The interviewer was female. |
| 5 | **Experience and training** | What experience or training did the researcher have? | Reported | Methods, 1.6 and 1.8 | Previous frontline long-term-care experience and online training in qualitative interviewing are described. |
| 6 | **Relationship established** | Was a relationship established before study commencement? | Reported | Methods, Sections 1.4 and 1.6 | The interviewer had no personal, family, caregiving, or other pre-existing relationship with the participants. Pre-interview contact was limited to eligibility screening, provision of study information, scheduling, and informed consent. |
| 7 | **Participant knowledge of interviewer** | What did participants know about the researcher? | reported | Methods, 1.4 | Before each interview, the interviewer introduced herself by name and informed participants of her affiliation with Hunan Normal University, her role as a master’s nursing student and interviewer, and that the study was conducted under the supervision of her academic supervisor. She then explained the study purpose, procedures, voluntary nature of participation, confidentiality protections, expected interview duration, and audio-recording arrangements. |
| 8 | **Interviewer characteristics** | What interviewer characteristics or assumptions were reported? | Reported | Methods, 1.6 | The interviewer’s background and possible influence on the interview and analysis are discussed, together with reflexive journaling. |
| 9 | **Methodological orientation and theory** | What methodological orientation underpinned the study? | Reported | Methods, 1.1 and 1.7 | Descriptive qualitative design and codebook-oriented thematic analysis are stated. COM-B was applied after inductive theme development. |
| 10 | **Sampling** | How were participants selected? | Reported | Methods, 1.2 | Five nursing homes in Hunan Province were purposively selected to reflect variation in ownership and service model, including public and private facilities with different service orientations. Eligible nursing assistants were subsequently recruited from these facilities. |
| 11 | **Method of approach** | How were participants approached? | Reported | Methods, 1.2 | Facility administrators distributed recruitment information containing the researcher’s telephone number. Interested nursing assistants contacted the researcher directly and were subsequently screened for eligibility and informed about the study. |
| 12 | **Sample size** | How many participants were in the study? | Reported | Methods, 1.2; Results | Fifteen nursing assistants were recruited. Three completed pilot interviews and were excluded from the analysis. The final analytic sample comprised 12 nursing assistants who completed the main-study interviews. |
| 13 | **Non-participation** | How many people refused or dropped out, and why? | Partially reported | Methods, 1.2 | A total of 15 nursing assistants were recruited.No participant withdrew after enrollment. |
| 14 | **Setting of data collection** | Where were the data collected? | Reported | Methods, 1.4 | Face-to-face interviews were conducted in a quiet, private room within the participating nursing homes. Telephone participants took the call alone in a private setting where they would not be interrupted or overheard. |
| 15 | **Presence of non-participants** | Was anyone else present besides the participant and researcher? | Reported | Methods, 1.4 | Face-to-face interviews were conducted with only the participant and interviewer present. Before each telephone interview, participants confirmed that they were alone in a private setting where they could not be interrupted or overheard. |
| 16 | **Description of sample** | What important sample characteristics were reported? | Reported | Methods, 1.2 and Table 1 | Sex, age, education, facility category and work experience are reported. |
| 17 | **Interview guide** | Were questions/prompts provided and was the guide pilot tested? | Reported | Methods, 1.3; Supplementary File 2 | Interview questions are listed and the guide was piloted with three eligible nursing assistants. |
| 18 | Repeat interviews | Were repeat interviews carried out? | Reported | Methods, 1.4 | No repeat interviews were conducted. |
| 19 | **Audio/visual recording** | Was audio or visual recording used? | Reported | Methods, 1.4 and 1.8 | Interviews were audio-recorded with consent. |
| 20 | **Field notes** | Were field notes made during or after interviews? | Reported | Methods, 1.5, 1.6 and 1.8 | Notes were taken during interviews and a reflexive journal was completed within 24 hours. |
| 21 | **Duration** | What was the duration of interviews? | Reported | Methods, 1.4 | Interviews lasted approximately 20–30 minutes. |
| 22 | **Data saturation** | Was data saturation discussed? | Reported | Methods, 1.2 | Two coders compared new codes and provisional themes after each formal interview; a third researcher contributed to the final assessment. Recruitment ended when successive interviews added no substantive new codes, relationships or thematic changes. |
| 23 | **Transcripts returned** | Were transcripts returned to participants for comment/correction? | Not undertaken | Methods, 1.8 | Full interview transcripts were not returned to participants for comment or correction. Participants were contacted only when clarification of an unclear statement was required. |
| 24 | **Number of data coders** | How many data coders coded the data? | Reported | Methods, 1.7 | Two researchers independently coded the transcripts and discussed disagreements with a third researcher. |
| 25 | **Description of coding tree** | Was a coding tree or coding framework described? | Reported | Methods, 1.7 | The progression from data segments to initial codes, subthemes, and themes is described in Section 1.7. Supplementary File 3 provides the thematic analysis codebook, including operational definitions, inclusion and exclusion criteria, data sources, and representative extracts. |
| 26 | **Derivation of themes** | Were themes identified in advance or derived from the data? | Reported | Methods, 1.7 | Themes were derived inductively from Chinese transcripts; COM-B was used after theme development. |
| 27 | **Software** | What software was used to manage the data? | Reported | Methods, 1.7 | NVivo 15.0 was used. |
| 28 | **Participant checking** | Did participants provide feedback on the findings? | Not undertaken | Methods, 1.8 | Preliminary findings were not returned to participants for feedback or validation. |
| 29 | **Quotations presented** | Were participant quotations used and identified? | Reported | Methods, 1.2 and 1.9; Results | Participant quotations are labelled N1–N12. Translation and back-translation procedures are described. |
| 30 | **Data and findings consistent** | Was there consistency between the data presented and the findings? | Reported | Results and Table 2 | Themes are supported by labelled quotations and subsequently mapped to COM-B domains. |
| 31 | **Clarity of major themes** | Were major themes clearly presented? | Reported | Abstract; Results; Discussion | Five major themes are named, described and illustrated with quotations. |
| 32 | **Clarity of minor themes/diverse cases** | Were minor themes or diverse cases discussed? | Reported | Results, Sections 2.1–2.5 | The Results describe variation in participants’ accounts, including enabling practices in better-resourced facilities, interdisciplinary collaboration in a small number of facilities, differences in residents’ responses to encouragement, and situations in which manageable risk and supervision supported independence-supportive care. |

**Reference**

Tong A, Sainsbury P, Craig J. Consolidated criteria for reporting qualitative research (COREQ): a 32-item checklist for interviews and focus groups. Int J Qual Health Care. 2007;19(6):349–357. doi:10.1093/intqhc/mzm042. PMID: 17872937.
