# Supplementary material for: Behavioral choices and enabling conditions for implementing independence-supportive care among nursing assistants in residential aged care facilities in Hunan Province, China: a qualitative study
Source: Front Public Health. 2026 Oct 6;14:1972560. doi: 10.3389/fpubh.2026.1972560 (PMC13682637; doi:10.3389/fpubh.2026.1972560)
Supplement: Supplementary file 2 [file Supplementary_file__2.docx]

**Supplementary File 2**

**Complete Bilingual Interview Guide**

Behavioral Choices and Enabling Conditions for Implementing Independence-Supportive Care Among Nursing Assistants in Residential Aged Care Facilities in Hunan Province, China: A Qualitative Study

The semi-structured interview guide was developed through literature review, research-team discussion, and pilot interviews. Follow-up prompts were used when clarification or elaboration was needed. The English and Chinese versions are presented below.

# English-Language Interview Guide

1. Have you heard of independence support or assistance for independence? How do you understand this concept?
2. Do you encourage functionally dependent older adults to do tasks that remain within their ability, such as eating or dressing by themselves?
3. What benefits may older adults gain from doing tasks within their ability?
4. Do you think encouraging older adults to do these tasks involves risks? What risks have you encountered?
5. What difficulties does this approach create for nursing assistants? Please give examples.
6. What are the main care needs of the older adults you currently support?
7. Do you support independence-supportive care? Why?
8. Under what circumstances do you encourage an older adult to do tasks within their ability?

# Chinese-Language Interview Guide

9. 您是否听说过“自立支援”或“自立照护”？您如何理解这一概念？
10. 对于生活功能受限的老年人，您是否会鼓励他们完成仍在能力范围内的事情，例如自己吃饭或穿衣？
11. 您认为老年人在能力范围内完成这些事情可能获得哪些益处？
12. 您认为鼓励老年人完成这些事情存在风险吗？您遇到过哪些风险？
13. 这种照护方式会给养老护理员带来哪些困难？请举例说明。
14. 您目前照护的老年人主要有哪些照护需求？
15. 您是否支持自立支援照护？为什么？
16. 在什么情况下，您会鼓励老年人完成能力范围内的事情？
