# Supplementary material for: Behavioral choices and enabling conditions for implementing independence-supportive care among nursing assistants in residential aged care facilities in Hunan Province, China: a qualitative study
Source: Front Public Health. 2026 Oct 6;14:1972560. doi: 10.3389/fpubh.2026.1972560 (PMC13682637; doi:10.3389/fpubh.2026.1972560)
Supplement: Supplementary file 3 [file Supplementary_file__3.docx]

# 2. Theme structure aligned with the Results

| **Results section** | **Theme title** | **Subtheme title(s)** |
| --- | --- | --- |
| 2.1 | Theme 1: Knowledge and Skills Related to Independence-Supportive Care Remain Unstandardized | 2.1.1 Independence-supportive care is already practiced informally, but nursing assistants’ competencies have not been standardized 2.1.2 Training emphasizes safety procedures but gives limited attention to functional improvement |
| 2.2 | Theme 2: Residents’ Functional Status, Willingness, and Expectations of Care Affect Their Participation | 2.2.1 Physical limitations and long-term dependence reduce residents’ willingness to participate 2.2.2 Expectations of full-service care and comparisons with other residents affect participation |
| 2.3 | Theme 3: Poor Integration Between Assessment and Care Planning Prevents Retained Abilities From Becoming Daily Care Goals | 2.3.1 Care-level classifications replace task-specific assessment 2.3.2 Formal assessments and rehabilitation plans are not adequately integrated into daily care |
| 2.4 | Theme 4: Limited Staffing, Time, and Assistive Devices Lead Nursing Assistants to Take Over Residents’ Tasks | 2.4.1 Staff shortages and competing tasks reduce the time available for support 2.4.2 Limited access to assistive devices increases the difficulty of care |
| 2.5 | Theme 5: Safety Risks and Accountability Pressures Promote Risk-Averse Care | No subtheme specified in the Results. |

## 2.1 Theme 1: Knowledge and Skills Related to Independence-Supportive Care Remain Unstandardized

| **Code** | **Results subtheme** | **Analytical code** | **Operational definition** | **Include when** | **Exclude when** | **Typical data sources** | **Representative translated data extract(s) reported in the Results** |
| --- | --- | --- | --- | --- | --- | --- | --- |
| K1 | 2.1.1 Independence-supportive care is already practiced informally, but nursing assistants’ competencies have not been standardized | Unfamiliarity with formal terminology | Use when a nursing assistant has not heard of independence-supportive care, misunderstands the term, or knows the practice but not its formal label. | Explicit statements of not knowing the term; interpreting it as another concept; recognizing the practice only after explanation. | Lack of knowledge about a particular device or clinical procedure without reference to the care concept. | N2, N4, N7, N9, N10 | “I’ve never heard of independence-supportive care. Does it mean volunteering?” (N2)  “I haven’t heard those formal terms before, but I know that moving around is good for older adults. If you always leave them in bed, they become less and less willing to move.” (N4) |
| K2 | 2.1.1 Independence-supportive care is already practiced informally, but nursing assistants’ competencies have not been standardized | Exposure through formal eldercare education | Use when principles similar to independence-supportive care were learned through school, professional education, or skills competitions. | Formal education or competitions that teach maintenance or improvement of residents’ abilities. | Learning only through coworkers, daily trial and error, or social media. | N3, N7, N8, N12 | “When I was in school, I regularly took part in skills competitions. We were always taught to encourage older adults to do as much as possible by themselves. That way, they could maintain their current physical abilities or gradually improve.” (N3) |
| K3 | 2.1.1 Independence-supportive care is already practiced informally, but nursing assistants’ competencies have not been standardized | Experience-based assessment of retained ability | Use when nursing assistants determine what residents can do through observation, informal trials, or accumulated care experience rather than a standardized task-specific assessment. | Letting a resident try; observing use of a spoon, limbs, or daily routines; gradually identifying ability. | Formal assessment conducted by an assessor, nurse, physician, or rehabilitation therapist. | N1, N2, N4, N6, N7, N10, N11 | “For residents with disabilities, I first let them try using a spoon by themselves. If they really can’t manage it, then I feed them. I also encourage them to move their limbs and joints.” (N6) |
| K4 | 2.1.1 Independence-supportive care is already practiced informally, but nursing assistants’ competencies have not been standardized | Peer discussion and informal learning | Use when care knowledge is developed through coworker communication and gradual familiarization with residents’ habits and preferences. | Peer discussion, shift-based knowledge exchange, and learning through day-to-day interaction. | Structured classroom teaching or documented competency assessment. | N1, N2, N5, N8 | “We talk with one another about the details of each resident’s care. We learn about their habits and preferences and gradually figure out the best way to care for them.” (N8) |
| K5 | 2.1.2 Training emphasizes safety procedures but gives limited attention to functional improvement | Safety and basic-care procedures dominate training | Use when training or examinations prioritize fall prevention, pressure injury prevention, transfers, emergency response, or basic activities of daily living. | Training content centred on safety, first aid, transfers, infection control, and routine care procedures. | Training explicitly focused on improving functional independence or graded self-care practice. | N1, N2, N4, N5, N6, N8, N10, N11 | “Our examination covered fall prevention, pressure injury prevention, wheelchair transfers, and stretcher transfers. We were expected to know most of these procedures.” (N2)  “Our department provides training every month and every year. There are so many procedures, such as wheelchair transfers and the Heimlich maneuver. Most of them are basic care procedures and first-aid techniques. There are so many that I can’t even remember all their names.” (N5) |
| K6 | 2.1.2 Training emphasizes safety procedures but gives limited attention to functional improvement | Reablement-oriented training as an enabling exception | Use when a facility does not necessarily use the same term but explicitly teaches reablement, rehabilitation, standing, walking, dressing, or self-feeding. | Training that links daily activities to retained ability, achievement, or functional recovery. | Routine assistance or safety training without a functional improvement goal. | N1, N3, N12 | “Our facility has discussed a similar concept called reablement. It means helping older residents demonstrate what they can still do at this stage of life and giving them a sense of achievement. It is very similar to independence-supportive care.” (N1) “What you described as independence-supportive care means allowing older residents to dress and stand by themselves. The rehabilitation services we provide include dynamic balance, standing balance, walking, dressing and undressing, and eating. These are covered in our facility’s training.”(N12) |

## 2.2 Theme 2: Residents’ Functional Status, Willingness, and Expectations of Care Affect Their Participation

| **Code** | **Results subtheme** | **Analytical code** | **Operational definition** | **Include when** | **Exclude when** | **Typical data sources** | **Representative translated data extract(s) reported in the Results** |
| --- | --- | --- | --- | --- | --- | --- | --- |
| R1 | 2.2.1 Physical limitations and long-term dependence reduce residents’ willingness to participate | Physical weakness and activity-related fatigue | Use when limited strength, prolonged disability, or fatigue reduces residents’ capacity or willingness to practise an activity. | Residents describe walking or activity as tiring; physical weakness restricts participation. | Refusal based mainly on payment expectations, comparison with peers, or staff workload. | N1–N12 | “The residents who come here are usually physically weak. When we ask them to walk regularly, some say it is too tiring and refuse. We can’t force them.” (N2) |
| R2 | 2.2.1 Physical limitations and long-term dependence reduce residents’ willingness to participate | Perceived futility of rehabilitation in old age | Use when residents believe that age or disability makes exercise or self-care useless. | Statements that training will not help because the resident is old or already disabled. | Temporary refusal caused by pain, dizziness, or an acute illness. | N3, N5, N12 | “Sometimes the residents themselves are the main barrier. They think they’re too old for these activities to make any difference, so they don’t want to do them.” (N3) |
| R3 | 2.2.1 Physical limitations and long-term dependence reduce residents’ willingness to participate | Habituated dependence after prolonged bed rest or assistance | Use when residents become accustomed to lying in bed or receiving assistance and subsequently resist resuming activity. | A prior ability or preference changes after prolonged dependence; residents no longer want to get up or act independently. | Complete inability caused by an irreversible physical condition. | N2, N5, N9, N10, N11, N12 | “There was an older woman who wasn’t used to lying in bed and insisted on sitting in a wheelchair. Later, she fractured her leg and couldn’t move or sit in the wheelchair. Now she has become used to staying in bed. When we ask her to sit in the wheelchair, she refuses and says she feels dizzy when she sits up. She has become so used to lying down that she no longer wants to get out of bed.” (N11) |
| R4 | 2.2.1 Physical limitations and long-term dependence reduce residents’ willingness to participate | Escalating resistance to repeated persuasion | Use when repeated encouragement irritates a resident or further reduces willingness to participate. | The nursing assistant repeatedly encourages an activity and the resident becomes annoyed or increasingly resistant. | A resident accepts encouragement or improves with graded goals. | N5 | “Some residents simply don’t want to do it because they think it’s too much trouble. I used to spend a lot of time encouraging one resident to walk more instead of always sitting in a wheelchair. But the more I talked about it, the more annoyed he became, so eventually I stopped.” (N5) |
| R5 | 2.2.1 Physical limitations and long-term dependence reduce residents’ willingness to participate | Participation dependent on prompting | Use when a resident performs an activity only after immediate reminders or encouragement and stops when prompting is absent. | Clear understanding or retained ability is present, but action depends on staff cues. | The resident is physically or cognitively unable to understand the prompt. | N1, N5, N9, N11 | “If a resident doesn’t want to move, there isn’t much we can do. We can keep talking to them, but that’s about it. We have one older man who understands everything clearly but can’t express himself well. He just doesn’t want to move. When we remind him, he moves a little. If we don’t remind him, he does nothing. We see all kinds of residents here.” (N9) |
| R6 | 2.2.2 Expectations of full-service care and comparisons with other residents affect participation | Family expectation of comprehensive task substitution | Use when family members view residential care as a service in which staff should complete all daily activities for the resident. | Families state that the resident was admitted so everything would be taken care of or ask staff to do more. | Families support exercise, reinforce goals, or request appropriately graded assistance. | N1, N2, N12 | “When you explain independence-supportive care to family members, they may say it isn’t necessary. They believe they sent the resident here so that everything would be taken care of.” (N1) “Family members usually want you to do more so that the resident can do less. I think that is probably the biggest issue.” (N12) |
| R7 | 2.2.2 Expectations of full-service care and comparisons with other residents affect participation | Paid-care expectations shape judgements of adequacy | Use when payment for institutional care leads residents or families to expect more visible staff work and to judge graded support as insufficient. | References to paying for care, expecting staff to perform tasks, or judging even good care as inadequate. | Requests for assistance that are proportionate to actual functional limitations. | N2, N9, N11, N12 | “For example, if this were my father and I couldn’t care for him properly at home, I would pay to have him cared for here. Ninety percent of families are satisfied with the care we provide, but a few still think it isn’t enough, even when the resident is being well cared for.” (N2) “Some residents simply don’t want to make the effort. They think, ‘I’ve already paid, so why should I have to do these things? The nursing assistants should do them for me.’ Then they refuse to cooperate.” (N9) |
| R8 | 2.2.2 Expectations of full-service care and comparisons with other residents affect participation | Comparison with residents receiving full assistance | Use when residents request feeding or other assistance after observing that other residents receive it. | Explicit comparison with peers and a request for equivalent staff assistance. | Requests based solely on the resident’s own symptoms or functional decline. | N11 | “Some residents see other people receiving help and start relying on us too. When we tell them to do something themselves, some will try, but others refuse even after we talk to them. They say, ‘I paid for this.’ Some see nursing assistants feeding other residents and then want to be fed too.” (N11) |

## 2.3 Theme 3: Poor Integration Between Assessment and Care Planning Prevents Retained Abilities From Becoming Daily Care Goals

| **Code** | **Results subtheme** | **Analytical code** | **Operational definition** | **Include when** | **Exclude when** | **Typical data sources** | **Representative translated data extract(s) reported in the Results** |
| --- | --- | --- | --- | --- | --- | --- | --- |
| A1 | 2.3.1 Care-level classifications replace task-specific assessment | Institutional admission interpreted as evidence of dependence | Use when admission to residential care is treated as proof that the resident cannot care for themselves. | Statements implying that self-caring residents would not have been admitted. | Evidence-based assessment of a particular task or retained function. | N5, N6, N9 | “If the residents here could take care of themselves, their families wouldn’t have sent them here.” (N5) |
| A2 | 2.3.1 Care-level classifications replace task-specific assessment | Care delivered according to assigned level | Use when the assessment category or care level determines routine care tasks and assistance. | Staff report following the classification recorded on the assessment form. | Flexible assistance based on real-time performance of a specific task. | N1, N6, N10, N12 | “We work according to the level listed on the assessment form. For example, if the physician and nurse classify a resident as requiring Level 1 care, we provide care according to that classification.” (N10) |
| A3 | 2.3.1 Care-level classifications replace task-specific assessment | Severe disability equated with complete staff takeover | Use when long-term bed rest or disability is described as requiring staff to do everything. | Global descriptions of total dependence without distinguishing retained task abilities. | Statements identifying a specific activity the resident can still perform. | N5, N6, N7, N9, N10, N11 | “The residents I care for have disabilities and have been bedridden for a long time. Our staff have to do everything for them.” (N6) |
| A4 | 2.3.2 Formal assessments and rehabilitation plans are not adequately integrated into daily care | Formal assessment and rehabilitation controlled by other professionals | Use when physicians, nurses, assessors, or rehabilitation therapists control formal assessment and rehabilitation decisions while nursing assistants mainly implement instructions. | Nursing assistants report doing what professionals tell them or being excluded from formal decision-making. | An integrated model in which nursing assistants contribute to goal setting and follow-up. | N1, N2, N4, N7, N8, N9, N10, N12 | “Our facility employs professional assessors, and physicians and nurses are responsible for residents’ rehabilitation. We do what they tell us to do. At most, we report whether a resident is cooperating.” (N8)  “I have an associate degree in eldercare services and obtained an assessor qualification last year, but assessments are still conducted by the eldercare services department. The physicians don’t listen to us when it comes to rehabilitation.” (N10) |
| A5 | 2.3.2 Formal assessments and rehabilitation plans are not adequately integrated into daily care | Daily observations communicated through informal handover | Use when nursing assistants identify retained abilities during care and pass the information to the next shift rather than incorporating it into a formal plan. | Observation during daily care followed by handover or informal communication. | A formally documented reassessment that automatically changes the rehabilitation plan. | N1, N2, N8, N10, N12 | “We assess residents’ self-care abilities based on our own experience. If we observe that a resident can complete an activity, we pass that information on to the next shift so they know the resident can do it independently.” (N12) |
| A6 | 2.3.2 Formal assessments and rehabilitation plans are not adequately integrated into daily care | Limited participation in rehabilitation planning | Use when nursing assistants have little or no involvement in discussions about rehabilitation goals, activities, or assistance levels. | Explicit statements of not participating or only carrying out other professionals’ plans. | Routine interdisciplinary meetings with shared follow-up responsibility. | N4, N6, N7, N8, N10 | “We are responsible for residents’ daily care, but we are not fully involved in discussions about their rehabilitation plans. The physicians are responsible for that.” (N8)  “We didn’t participate in that program at all.” (N6) |
| A7 | 2.3.2 Formal assessments and rehabilitation plans are not adequately integrated into daily care | Communication routed primarily through families | Use when nursing assistants may communicate with rehabilitation staff, but the rehabilitation plan is mainly discussed with the family. | The family is the principal recipient of plan-related communication. | Nursing assistants and rehabilitation staff jointly revise daily activity goals. | N1 | “When we notice changes in a resident’s physical condition, we communicate with the rehabilitation therapist. However, the therapist mainly discusses the rehabilitation plan with the family.” (N1) |
| A8 | 2.3.2 Formal assessments and rehabilitation plans are not adequately integrated into daily care | Rehabilitation extended into daily activities | Use when nursing assistants reinforce rehabilitation gains during ordinary eating, dressing, mobility, or other daily care. | Professional assessment and planning are followed by practice embedded in daily routines. | Rehabilitation remains confined to scheduled therapy sessions. | N1, N3, N7 | “The rehabilitation therapist assesses each resident and develops an individualized rehabilitation plan. Our role is to support the exercises during daily activities and help residents complete tasks they were previously unable to perform.” (N7) |
| A9 | 2.3.2 Formal assessments and rehabilitation plans are not adequately integrated into daily care | Integrated social work, rehabilitation, and care model | Use when social workers, rehabilitation staff, and nursing assistants meet regularly and jointly follow residents’ progress. | Regular interdisciplinary discussion and coordinated follow-up. | Occasional one-way communication or isolated professional work. | N3, N7 | “We use an integrated model involving social work, rehabilitation, and care. The three groups meet regularly to discuss each resident’s situation and plan follow-up.” (N3) |

## 2.4 Theme 4: Limited Staffing, Time, and Assistive Devices Lead Nursing Assistants to Take Over Residents’ Tasks

| **Code** | **Results subtheme** | **Analytical code** | **Operational definition** | **Include when** | **Exclude when** | **Typical data sources** | **Representative translated data extract(s) reported in the Results** |
| --- | --- | --- | --- | --- | --- | --- | --- |
| W1 | 2.4.1 Staff shortages and competing tasks reduce the time available for support | Resident-led care requires additional time and energy | Use when allowing residents to complete an activity is slower and more labour intensive than staff takeover. | Direct comparison between quick staff completion and slow resident performance. | Extra time caused solely by an acute medical procedure. | N3, N4, N9, N11, N12 | “We can finish bathing a resident very quickly if we do it ourselves. When residents bathe themselves, they move slowly, so it takes much more of our time and energy.” (N4) |
| W2 | 2.4.1 Staff shortages and competing tasks reduce the time available for support | Staff shortages and competing basic-care demands | Use when workload and staffing ratios leave insufficient capacity for psychological support or independence-supportive practice. | One worker cares for many residents and reports exhaustion or inability to attend to additional needs. | A resident refuses despite adequate staffing and time. | N2, N4, N5, N7, N9, N10, N12 | “We are short-staffed, and one person has only so much energy. Caring for seven or eight residents is already exhausting.” (N9) |
| W3 | 2.4.1 Staff shortages and competing tasks reduce the time available for support | Reduced frequency and rotation of practice opportunities | Use when daily practice is replaced by weekly or rotating opportunities because staff cannot support everyone. | Activities are scheduled less often or residents take turns because of workload. | A clinically prescribed low-frequency programme unrelated to staffing. | N2, N10 | “We care for a lot of residents. I’m responsible for six. It isn’t realistic to help them practice walking every day. At most, we do it once a week and rotate among residents.” (N10) |
| W4 | 2.4.2 Limited access to assistive devices increases the difficulty of care | Facility cost limits provision of assistive devices | Use when facilities do not provide devices because universal provision is considered too expensive. | Cost is explicitly linked to non-provision and families are advised to buy devices. | A device is available but the resident chooses not to use it. | N4, N7, N9, N10, N12 | “Our facility doesn’t provide these devices, but we recommend that families purchase them. If the facility had to provide everything, the cost would be very high.” (N12) |
| W5 | 2.4.2 Limited access to assistive devices increases the difficulty of care | Basic provision supplemented by loan, rental, or family purchase | Use when a limited basic supply exists, while special devices depend on borrowing, rental, or family purchase. | Descriptions of temporary loans, rental services, or out-of-pocket purchase. | Routine personal belongings unrelated to functional assistance. | N7, N9, N12 | “We have some assistive devices and provide the basic ones. For residents with special needs, we may offer a temporary loan or rental service. If we don’t have what they need, the family has to purchase it.” (N9) |
| W6 | 2.4.2 Limited access to assistive devices increases the difficulty of care | Manual assistance substitutes for unavailable equipment | Use when nursing assistants rely on relatives or coworkers to lift or transfer a resident because adequate support is unavailable. | Requests for physical help from family members or coworkers during transfer. | Team assistance that is already part of a planned two-person transfer protocol. | N2, N4, N6, N10, N11 | “If we can’t move a resident and the resident’s son is there, we ask him to help. Sometimes coworkers help one another.” (N6) |

## 2.5 Theme 5: Safety Risks and Accountability Pressures Promote Risk-Averse Care

| **Code** | **Results subtheme** | **Analytical code** | **Operational definition** | **Include when** | **Exclude when** | **Typical data sources** | **Representative translated data extract(s) reported in the Results** |
| --- | --- | --- | --- | --- | --- | --- | --- |
| S1 | No subtheme specified in the Results | Supervised autonomy within a controllable range | Use when nursing assistants support independent activity only when they can remain nearby and judge the activity manageable. | The resident acts within staff sight or ability to manage; assistance is available if needed. | Unsupervised activity with unclear risk or complete staff takeover. | N1, N2, N4, N7, N8, N10, N11 | “If the resident is within my sight and is practicing something they can manage independently, I think that is a good thing.” (N8)  “I’m willing to let residents be active as long as it is within my ability to manage. If the activity is too difficult, I put safety first.” (N11) |
| S2 | No subtheme specified in the Results | Safety-first restriction of independent activity | Use when a high perceived fall or injury risk leads staff to limit or prevent independent activity. | The activity is supported at low risk but restricted when risk is high. | The resident refuses for reasons unrelated to safety. | N1, N4, N7, N9, N10, N11, N12 | “Our facility emphasizes safety above everything else. If a resident can use the toilet independently, I support that. But if there is a high risk of falling, I don’t.” (N1) |
| S3 | No subtheme specified in the Results | Fear of financial penalties following an adverse event | Use when possible pay deductions or other financial consequences make nursing assistants more cautious. | Explicit reference to money being deducted or financial punishment after something goes wrong. | Concern about resident harm without reference to staff consequences. | N6 | “We do want residents to move more. The more they move, the less likely their abilities are to deteriorate. But we worry that if something goes wrong, money will be deducted from our pay.” (N6) |
| S4 | No subtheme specified in the Results | Personal responsibility for accidental injury | Use when nursing assistants restrict activity because they expect to bear responsibility for an injury. | Explicit reference to responsibility, blame, or accountability for an accident. | General safety awareness without personal accountability concerns. | N2, N6, N9, N12 | “I still don’t dare let residents move around by themselves. If they are accidentally injured, we are the ones who have to take responsibility.” (N9) |
| S5 | No subtheme specified in the Results | Preference for low-risk, controllable care | Use when nursing assistants prefer bedridden care because it reduces fall risk and makes tasks more predictable. | Bedridden residents are described as easier or safer because mobility risks are absent. | Bedridden care selected solely because of clinical need, without comparison to risk. | N9, N10, N11, N12 | “I prefer caring for residents who are bedridden because at least there is no risk of them falling. With bedridden residents, the main tasks are preventing pressure injuries, helping with meals, and providing personal hygiene.” (N12) |
